# Validation and utilization of an internally controlled multiplex Real-time RT-PCR assay for simultaneous detection of enteroviruses and enterovirus A71 associated with hand foot and mouth disease

**DOI:** 10.1186/s12985-015-0316-2

**Published:** 2015-06-09

**Authors:** Tran Tan Thanh, Nguyen To Anh, Nguyen Thi Tham, Hoang Minh Tu Van, Saraswathy Sabanathan, Phan Tu Qui, Tran Thuy Ngan, Tran Thi My Van, Lam Anh Nguyet, Nguyen Thi Han Ny, Le Thi My Thanh, Ong Kien Chai, David Perera, Do Chau Viet, Truong Huu Khanh, Do Quang Ha, Ha Manh Tuan, Kum Thong Wong, Nguyen Thanh Hung, Nguyen Van Vinh Chau, Guy Thwaites, H Rogier van Doorn, Le Van Tan

**Affiliations:** Oxford University Clinical Research Unit in partnership with the Hospital for Tropical Diseases, Wellcome Trust Major Overseas Programme, Ho Chi Minh City, Vietnam; Hospital for Tropical Diseases, Ho Chi Minh City, Vietnam; Children Hospital Number One, Ho Chi Minh City, Vietnam; Children Hospital Number Two, Ho Chi Minh City, Vietnam; Facuty of Medicine, University of Malaya, Lumpur, Malaysia; Institute of Health and Community Medicine, Universiti Malaysia Sarawak, Sarawak, Malaysia; Centre for Tropical Medicine, Nuffield Department of Medicine, University of Oxford, Oxford, UK

**Keywords:** Hand foot and mouth disease, Enteroviruses, Enterovirus A71, Real-time RT-PCR, Diagnosis

## Abstract

**Background:**

Hand foot and mouth disease (HFMD) is a disease of public health importance across the Asia-Pacific region. The disease is caused by enteroviruses (EVs), in particular enterovirus A71 (EV-A71). In EV-A71-associated HFMD, the infection is sometimes associated with severe manifestations including neurological involvement and fatal outcome. The availability of a robust diagnostic assay to distinguish EV-A71 from other EVs is important for patient management and outbreak response.

**Methods:**

We developed and validated an internally controlled one-step single-tube real-time RT-PCR in terms of sensitivity, linearity, precision, and specificity for simultaneous detection of EVs and EV-A71. Subsequently, the assay was then applied on throat and rectal swabs sampled from 434 HFMD patients.

**Results:**

The assay was evaluated using both plasmid DNA and viral RNA and has shown to be reproducible with a maximum assay variation of 4.41 % and sensitive with a limit of detection less than 10 copies of target template per reaction, while cross-reactivity with other EV serotypes was not observed. When compared against a published VP1 nested RT-PCR using 112 diagnostic throat and rectal swabs from 112 children with a clinical diagnosis of HFMD during 2014, the multiplex assay had a higher sensitivity and 100 % concordance with sequencing results which showed EVs in 77/112 (68.8 %) and EV-A71 in 7/112 (6.3 %). When applied to clinical diagnostics for 322 children, the assay detected EVs in throat swabs of 257/322 (79.8 %) of which EV-A71 was detected in 36/322 (11.2 %) children. The detection rate increased to 93.5 % (301/322) and 13.4 % (43/322) for EVs and EV-A71, respectively, when rectal swabs from 65 throat-negative children were further analyzed.

**Conclusion:**

We have successfully developed and validated a sensitive internally controlled multiplex assay for rapid detection of EVs and EV-A71, which is useful for clinical management and outbreak control of HFMD.

**Electronic supplementary material:**

The online version of this article (doi:10.1186/s12985-015-0316-2) contains supplementary material, which is available to authorized users.

## Introduction

Hand foot and mouth disease (HFMD) is a commonly benign and self-limiting viral infection of infants and young children worldwide. The disease is caused by acute infection with *Enterovirus A* of the family *Picornaviridae* including coxsackievirus A6 (CV-A6), CV-A10, CV-A16, and enterovirus A71 (EV-A71) [1–5]. However, in contrast to the common mild patterns of previous sporadic epidemics, since 1997 fulminant outbreaks of HFMD involving millions of children with neurological involvement and sometimes fatal cardiopulmonary complications have occurred across the Asia-Pacific region with EV-A71 being the most frequent pathogen isolated from patients with clinical complications and fatal cases [6–9]. Despite the public health burden of (EV-A71-associated) HFMD, currently, no clinically proven effective antiviral agents or vaccines are available for routine use.

The availability of a rapid, high-throughput and accurate diagnostic assay that can simultaneously detect and distinguish between EVs and EV-A71 is an ideal aid to patient management and may help outbreak response with regards to predicting the possible level of severity of the outbreak and thereby initiating appropriate public health interventions. Although EVs/EV-A71 diagnosis can be achieved by virus isolation, cell culture based methods are time-consuming and insensitive [10]. Molecular assays based on specific amplification viral nucleic acids, in particular real-time PCR, are sensitive, specific and high-throughput and are therefore methods of choice [11, 12]. Herein, we describe the development and validation of an internally controlled single-step multiplex real-time RT-PCR for simultaneous detection of EVs and EV-A71 in clinical specimens from patients with HFMD.

## Results

### Analytical specificity and 95 % LLOD

The analytical specificity of the assay was evaluated on extracted RNA derived from EV strains belonging to 9 different EV serotypes and isolates belonging to 8 different (sub)genogroups of EV-A71 (Table 1). The fluorescent signal of the EV probe was detected from all reactions containing viral RNA extracted from diverse EV serotypes belonging to different species including A (including EV-A71), B and C, whereas the signal of the EV-A71 probe was detected only from reactions containing viral RNA extracted from EV-A71 of all 8 tested subgenogroups, but not from other EV A-C types.

**Table 1:** Virus isolate and origin/source

Strain	Serotypes	Species	Subgeno-group	Accession number	Location/source	Year of isolation
9522	EV-A71	A	C1	AY258300	Malaysia	2003
8 M/6/99	EV-A71	A	C2	AY126012	Australia	1999
001-KOR-00	EV-A71	A	C3	AY125966	Korea	2000
VN152	EV-A71	A	C4	NA	Vietnam	2011
VN5559	EV-A71	A	C4	AM490152	Vietnam	2005
VN5784	EV-A71	A	C5	AM490158	Vietnam	2005
13903	EV-A71	A	B3	AY207648	Malaysia	1997
A10/4	EV-A71	A	B4	AF376067	Malaysia	2000
18431	EV-A71	A	B5	NA	Malaysia	2006
H08214350	CV-A4	A	NA	NA	^a^	NA
H07314334	CV-A16	A	NA	NA	^a^	NA
H06418058	CV-A21	C	NA	NA	^a^	NA
H06474277	E-6	B	NA	NA	^a^	NA
H08306574	E-30	B	NA	NA	^a^	NA
H05062273	CV-B5	B	NA	NA	^a^	NA
Gdula	CV-A6	A	NA	AY42164	^b^	NA
Texas 12	CV-A12	A	NA	AY42168	^b^	NA
G-14	CV-A14	A	NA	AY421769	^b^	NA

Note: ^a^Public Health England, UK, ^b^American Type Culture Collection, *NA*: Not available

The analytical sensitivity (95 % LLOD) of the assay was evaluated using both plasmid DNA and EV-A71 RNA. The detection limit of the assay for EVs and EV-A71 was 7.1 and 5.3 copies of plasmid DNA per reaction, and was 2.9 and 1.1 TCID_50_ (equivalent to 9.1 and 3.2 cDNA copies per reaction, respectively) of EV-A71, respectively.

### Linearity and precision

**Fig. 1:**
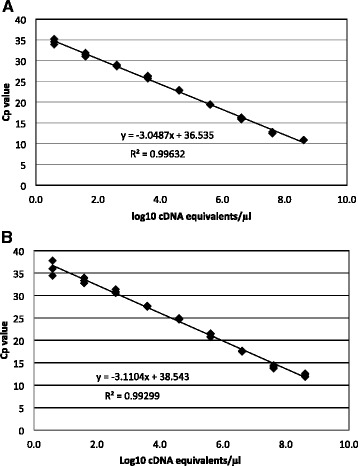
Linearity of the multiplex RT-PCR assay using 10-fold dilution series of plasmid DNA containing the PCR templates of EV (**a**) and EV-A71 (**b**), Cp = crossing point

**Fig. 2:**
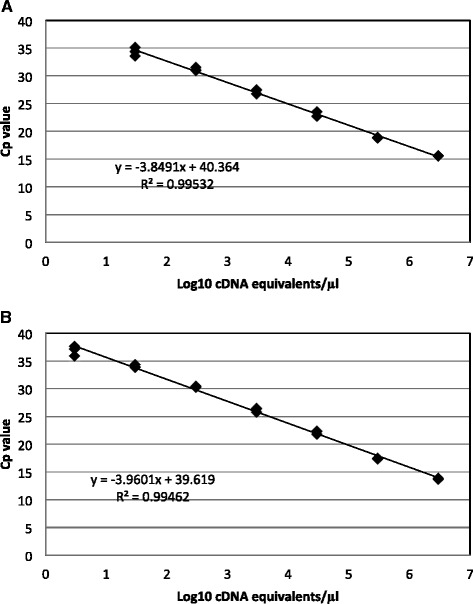
Linearity of the multiplex RT-PCR assay using 10-fold dilution series of RNA extracted from an EV-A71 isolate; EV (**a**) and EV-A71 (**b**), Cp: crossing point. Concentration used were equivalent to 3 × 10^1^ to 3 × 10^6^ cDNA copies per reaction for EV and 3 × 10^0^ to 3 × 10^6^ cDNA copies per reaction for EV-A71

The linearity of the multiplex assay was calculated from amplification of serial ten-fold dilutions of plasmid DNA and EV-A71 RNA. The linear regression of plasmid DNA and viral RNA was 0.993 and 0.995, and 0.996 and 0.995 for the EV and EV-A71, respectively (Figs. 1 and 2).

The precision of the assay was assessed by measuring variations within (intra-assay variation) and between runs (inter-assay variation) at high, medium, and low concentrations of template. The maximum intra- and inter-assay variations using plasmid DNA and viral RNA were 3.12 % and 4.41 %, respectively (Table 2).

**Table 2:** Maximum intra and inter assay variations in different concentrations of plasmid DNA and viral RNA

Concentration		Plasmid DNA (copies)			Viral RNA (TCID_50_)		
		4 × 10^6^	4 × 10^3^	4 × 10^1^	9.6 × 10^5^	9.6 × 10^3^	9.6 × 10^1^
Maximum EV assay co-variation	Intra-assay	0.53	0.93	1.37	1.49	0.84	0.77
	Inter-assay	3.13	1.36	2.18	4.41	3.01	2.54
Maximum EV-A71 assay co-variation	Intra-assay	0.52	1.00	2.18	1.34	0.73	0.73
	Inter-assay	3.19	1.37	1.85	2.66	1.56	1.53

### Comparison with nested RT-PCR and sequencing confirmation

**Fig. 3:**
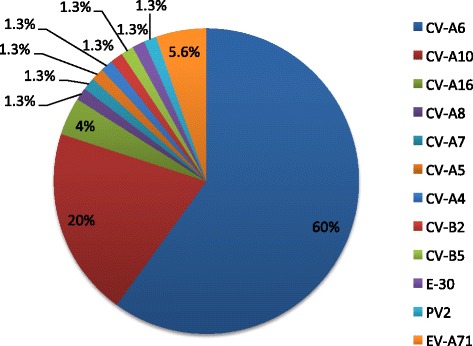
Pie chart showing the frequency of specific EV serotypes detected by nested RT-PCR followed by sequencing of amplified products; CV-A6 (n = 45), CV-A 10 (15), CV-A 16 (3), EV-A71 (4), CV-A 8 (1), CV-A 7 (1), CV-A 5 (1), CV-A 4 (1), CV-B2 (1), CV-B5 (1), E-30 (1), PV2 [vaccine strain] (1)

To assess the clinical specificity of the assay, a subset of 112 swabs (45 throat and 67 rectal swabs from 112 children with clinically suspected HFMD) was randomly selected for comparison with a published nested RT-PCR assay [13]. Among 102 specimens positive for EVs using multiplex real-time RT-PCR, 21 were not detected by the nested RT-PCR assay, while none of the 10 multiplex RT-PCR negative specimens were positive by the nested RT-PCR assay (Table 3). Further confirmation for the accuracy of the multiplex RT-PCR results was achieved by sequencing of amplified products generated by the VP1 assay. Of the 81 nested RT-PCR positive specimens, VP1 sequences were successfully obtained from 75 (71 non-EV-A71 and 4 EV-A71). Sequence analyses revealed that all 71 viruses detected by the EVs generic primers/probe but not by the EV-A71 assay belonged to 11 different EV serotypes of which CV-A6 accounted for 60 %, whereas all 4 EV-A71 positive specimens detected by the multiplex assay were confirmed as EV-A71 (Fig. 3).

**Table 3:** Comparison with VP1 nested RT-PCR and serotype confirmation by sequencing

Tests		Multiplex real-time RT-PCR			Total (n)
		EVs positive		EVs negative	
		Non-EV-A71	EV-A71		
Nested RT-PCR	Positive (n)	77	4	0	81
	Negative (n)	18	3	10	31
	Total (n)	95	7	10	112
VP1 sequencing (n)		71	4	0	75

### Assay application in clinical studies

The assay was applied in clinical samples (rectal and throat swabs) obtained from 322 children enrolled in our on-going HFMD research program. Multiplex RT-PCR analysis of throat swab specimens revealed an overall detection rate of 79.8 % (257/322) for EVs, of which EV-A71 was detected in 11.2 % (36/322). Further diagnostic effort on rectal-swab specimens from 65/322 (20.2 %) children whose throat swabs were negative by the multiplex assay showed that 44/65 (56.9 %) rectal swabs were EVs positive, of which 7(10.8 %) were EV-A71. No virus was detected in 21 paired throat and rectal swabs, corresponding to 6.5 %. Thus, the overall detection rates of EVs and EV-A71 in 322 clinically suspected HFMD children were 93.5 % (301/322) and 13.4 % (43/322), respectively.

## Discussion

We have successfully developed and validated a single-tube multiplex one-step real-time RT-PCR for high-throughput, sensitive, and specific detection of EVs and EV-A71 in clinical samples. The performance of the assay is internally controlled by adding an optimal amount of EAV to the tested specimens allowing for quality assessment of the whole procedure from nucleic acid extraction to amplification. Furthermore, this assay is rapid (turnaround time of less than 3 h), cost-effective (reagent associated cost of $ 8 per sample) and resource efficient (both EVs and EV-A71 are tested simultaneously) and is therefore useful for clinical management and outbreak response of HFMD.

Although primers and probes used in this study were derived from previous studies [8, 14, 15], different reporters and quenchers were selected and combined to ensure efficiency of assay performance (i.e. no cross-fluorescence). In addition, because when combined with the new reporter (Cy5) and quencher (BHQ3), the original EVs probe [14] produced a high baseline signal. The sequence of the EVs probe was therefore changed into its reverse complement to reduce high baseline signal (data not shown), while maintaining sensitivity and specificity as shown by the outcome of the specificity experiments.

We originally developed the EV-A71 assay that was successfully used for diagnosis of HFMD caused by EV-A71 in previous studies [8] and during the outbreak of severe HFMD in Cambodia in 2012 [16]. However, this assay was specifically designed for the detection of subgenogroups C4 and C5 that were circulating at that time [8, 16]. Our recent assessment of EV-A71 sequences available in the GenBank suggested that modification by incorporation degenerated bases in the primer sequences was required to ensure a detection of all EV-A71 subgenogroups (Additional file 1: Figure S1). Indeed, the EV-A71 assay with modified primers was able to detect EV-A71 isolates of subgenotypes B3–B5 and C1–C5 that have been circulating in the Asia-Pacific region, while maintaining specificity. Although the assay has not been evaluated for subgenotypes B1 and B2, *in silico* analysis showed that the primers and probes used in this assay would enable their detection (Additional file 1: Figure S1).

The multiplex RT-PCR assay has been fully evaluated in terms of linearity, analytical sensitivity, precision and specificity. When evaluated on a wide range of template concentrations, the assay showed a good analytical linearity, thus fulfilling the requirements of a clinical diagnostic molecular assay [17]. The 95 % LLOD was less than 10 copies RNA/DNA per reaction, and thereby compliant with MIQE (Minimum Information for publication of Quantitative real-time PCR Experiments) guidelines [18].

All tested EV serotypes, representing a broad range of types belonging to EV A-C were detected by the EVs generic PCR, which is in accordance with the published report [14]. The EV-A71 primer-probe was specific for EV-A71 viruses since cross-reactivity with other EV serotypes was not observed when the assay was tested on cultured isolates of different non-EV-A71 serotypes.

The assay performance was further compared against a VP1 nested RT-PCR by testing both assay performance on 112 rectal and throat swabs from 112 children with HFMD. The VP1 nested RT-PCR was selected as an assay for comparison because: 1) it can function as a ‘reference’ assay for assessment of our assay performance in terms of both sensitivity and specificity as it also allows for serotype determination of HFMD pathogens by sequencing of the obtained PCR amplicon [13], 2) it is a WHO recommended assay for diagnostics of HFMD patients [19], and 3) it has been widely implemented in diagnostic laboratories [20, 21]. In these clinical specimens, the assay described here was more sensitive than the VP1 nested RT-PCR. Indeed, the multiplex RT-PCR could detect the presence of EVs RNA in 102 specimens as compared to 81 by the nested one. In addition, the specificity of the diagnostic result was confirmed by VP1 sequencing of 71 specimens that were non-EV-A71 PCR positive and 4 EV-A71 positive specimens, which further confirms our initial sequence analyses including BLAST and sequence alignment comparison (data not shown) that showed not potential cross reactivity of EV-A71 assay with other EV serotypes.

Following our proposed diagnostic workflow, the assay was applied on samples from 322 children enrolled to our on-going HFMD studies between May and September 2014. Using throat swab alone for initial diagnosis, 80 % (257/322) of the children had EVs/EV-A71 detected in throat swabs. The detection rate increased with 13.5 % (i.e. an overall diagnostic rate of 93.5 % (301/322) was achieved) when rectal swabs from 65 patients who had a negative throat swab test were further analyzed, indicating that the use of throat swab alone would result in false negative results for a substantial proportion of cases.

It is worth to note that throat and rectal swabs have been used for diagnostics of HFMD patients, although throat swab is more sensitive by virus isolation [19, 22]. However, parallel testing of both throat and rectal swabs would not be cost-effective. Therefore, following our proposed flowchart, the rate of EVs/EV-A71 detection was maximized, while maintaining cost-effectiveness (i.e. only 20 % of the rectal swabs needed to be tested), and retaining the usefulness of throat swabs for subsequent analyses (such as virus isolation [22]).

Although detection and differentiation of EV-A71 can be achieved with the existing real-time RT-PCR assays [11, 12, 14, 23–29], some of these assays are not multiplexed, targeting either EVs [14, 29] or EV-A71 [12, 24, 26, 27] separately. Multiplex RT-PCRs for EV-A71 and other EVs have been recently developed [11, 23, 25, 28]. However, these assays did not include internal controls [11, 25, 28] or were not fully validated in terms sensitivity, linearity, precision, and specificity. Although a comparison of our assay with other published methods is desirable, the absence of a true gold standard limits the usefulness of this. It should however be noted that the 95 % LLOD of our assay was less than 10 copies per reaction, in agreement with MIQE guidelines (as mentioned above) and within the detection limit ranges of between 5 and 90 copies per reaction of those previously published assays. Our paper represents another option for internally controlled rapid and simultaneous detection of EVs and EV-A71.

## Conclusions

We have successfully developed and validated a single-tube multiplex one-step real-time RT-PCR for high-throughput, sensitive, and specific detection of EVs and EV-A71 in clinical samples, which can be a great aid to clinical management and outbreak response of HFMD in the future.

## Materials and methods

### Clinical samples

Diagnostic throat and rectal swab specimens used in this study were collected prospectively from 434 children with a clinical diagnosis of HFMD presenting or admitted to three referral hospitals for southern Vietnam in Ho Chi Minh City: Hospital for tropical Diseases (HTD) and Children’s Hospital 1 (CH1) and 2 (CH2), between May and September 2014. Swabs were collected in viral transport medium, divided into three aliquots and stored at -80 °C until analysis.

### Virus isolates

An EV-A71 subgenogroup C4 isolate from a child with HFMD admitted to HTD during the 2011 outbreak and 17 other different isolates belonging to different EV-A71 subgenogroups and non-EV-A71 serotypes obtained from the Department of Biomedical Science, University of Malaya, Malaysia, Public Health England, UK and American Type Culture Collection, USA were used for assay validation (Table 1).

Infective titers of 50 % tissue culture infectious doses (TCID_50_) were titrated on human rhabdomyosarcoma cells using a standard method [22]. The concentration of viral stock was converted to cDNA copies per μl by the multiplex real-time RT-PCR assay with plasmid DNA serving as standard control.

Equine Arteritis Virus (EAV) was used as an internal control and was prepared as described previously [15, 30]. The concentration of internal control virus was selected using RT-PCR analysis of serial tenfold dilutions and the amount of virus used in the PCR assay had expected crossing point (Cp) values of between 30 and 33 [15, 30].

### Primers and probes

Primers and probes for EVs, EV-A71 and EAV assays used in this study were adapted from previous studies [8, 14, 15, 31] with slight modifications and are listed in Table 4. Briefly, for the EVs assay, the original probe was replaced by its reverse-complement. Modification of EV-A71 primers and probe was done by incorporation of degenerate bases to allow detection of all known subgenotypes of EV-A71.

**Table 4:** Primer and probe sequences and concentration used in single reaction

Name	Sequence (5’→3’)	Final concentration	Note
EAV-F primer	CATCTCTTGCTTTGCTCCTTAG	400 nM	Internal control [15]
EAV-R primer	AGCCGCACCTTCACATTG	400 nM	
EAV-probe	FAM-CGCTGTCAGAACAACATTATTGCCCAC-BHQ1	100 nM	
ENT-F	CCCTGAATGCGGCTAAT	400 nM	Enterovirus specific primers and probe [14]
ENT-R	ATTGTCACCATAAGCAGCC	400 nM	
ENTr-probe	Cy5-ACCCAAAGTAGTCGGTTCCG -BHQ3	200 nM	
EV-A71-634 F	GGAGAACACAARCARGAGAAAGA	400 nM	Enterovirus 71 specific primers and probe [8]
EV-A71-743R	ACYAAAGGGTACTTGGAYTTVGA	400 nM	
EV-A71-probe	Cyan500-TGATGGGCACDTTCTCRGTGCG-BHQ1	40 nM	

Note: FAM = Carboxyfluorescein; *Cy5* = Cyanine 5; Cyan500 = Cyan 500 NHS ester; *BHQ* = black hole quencher; R = A and G; Y = T and C; V = A, C, and G; D = A, G, and T

### Nucleic acid extraction and RT-PCR

Viral RNA was extracted from clinical specimens and culture materials using the QIAamp Viral RNA Mini kit (QIAgen GmbH, Hilden, Germany). In brief, 140 μl of culture supernatant/throat/rectal swabs in viral transport medium was first mixed with 20 μl of EAV and total nucleic acid was then extracted according to the manufacturer’s instruction, eluted in 100 μl elution buffer and stored at -80 °C until used.

Real-time RT-PCR was done using the SuperScript^R^ III One-Step qRT-PCR System with Platinum^R^ Taq DNA Polymerase (Invitrogen, Carlsbad, CA, USA) and was performed in a LightCycler 480 II machine (Roche Diagnostics GmbH, Mannheim, Germany). The reaction was performed in a final volume of 25 μl containing 12.5 μl 2X RT-PCR reaction Mix, primers and probes at appropriate concentrations (Table 4), 0.5 μl Enzyme Mix, and 2 μl viral RNA or plasmid DNA. The cycling conditions included one cycle of 60 °C for 3 min, followed by 15 min at 53 °C and 2 min at 95 °C, and 45 cycles of 15 s at 95 °C, 1 min at 53 °C (including fluorescence acquisition) and 15 s at 72 °C.

### Preparation of plasmid controls

Amplified PCR products generated by RT-PCR amplification of viral RNA derived from an EV-A71 subgenogroup C4 isolate mentioned above utilizing EV and EV-A71 specific primers (Table 1) were purified and cloned into a pCR 2.1-TOPO plasmid using the pCR 2.1-TOPO TA Cloning kit (Invitrogen), following the manufacturer’s instruction. Constructed plasmid containing amplified product was purified using the QIAprep Spin Miniprep Kit (Qiagen). Plasmid concentration was determined by NanoDrop spectrometer (Thermo-Scientific, Loughborough, UK). An equal amount of plasmids containing the cloned EV and EV-A71 target genes was mixed together and stored at -80 °C until analysis.

### Lower limit of 95 % detection, linearity and precision

Analytical evaluation of the multiplex assay was done as previously described [17, 18] using both plasmid DNA controls and extracted RNA from an EV-A71 isolate (VN152, Table 1).

To obtain the lower limit of 95 % detection (95 % LLOD), the mixed EV/EV-A71 plasmid DNA controls were serially diluted two-fold to obtain a series of concentrations ranging from 20 copies/μl to 0.6 copies/μl. Dilution series of extracted viral RNA from an EV-A71 virus with concentrations ranging from 0.3-9.6 TCID_50_/μl equivalent to 0.9 – 30.1 cDNA copies/μl were used. The experiment was performed on these dilution series once a day on five consecutive days with 10 replicates for each concentration. The 95 % LLOD was estimated using the Probit regression model in SPSS 19 (SPSS Inc, NY, USA) as described previously [15].

The linearity was assessed by performing the assay on 10-fold serial dilutions of plasmid DNA controls (ranging from 10^8^ to 10^1^ copies/μl) or EV-A71 RNA (ranging from 9.6 × 10^5^ to 9.6 TCID_50_ /μl equivalent to 3.1 × 10^6^ to 30.1 cDNA copies/μl). For each material, the experiment was carried out in a single run with 5 replicates for each dilution point. Linearity was calculated using linear regression analysis in Microsoft Excel and a range of input with an R^2^ value of 0.99 was accepted.

The precision was determined using both plasmid DNA and viral RNA controls at high, intermediate and low concentrations. The experiments were performed daily on five consecutive days in quintuplicate for each concentration. The intra- and inter-assay variability were calculated by assessing the Cp value deviation.

### Assay interpretation

The procedure was monitored by including an optimal amount of EAV (Cp value between 30 and 33) functioning as internal control for nucleic acid extraction and amplification steps. A sample was considered positive if negative controls were negative and the tested sample was positive with a Cp value equal to or less than 40. A sample was considered negative if negative controls were negative, positive controls were positive with Cp values of about 30 and internal control showed a positive result with a Cp value between 30 and 33. A result was considered as inconclusive if negative controls were positive and/or the internal control showed a result outside of the expected range and no amplified signal was observed for EVs/EV-A71 [30].

### Nested RT-PCR, sequencing and sequence analysis

The nested RT-PCR targeting viral protein 1 (VP1) gene sequence was carried out as previously described [13], and served as a reference for evaluation of the multiplex RT-PCR as there is currently no gold standard assay for diagnosis of HFMD. For this purpose, 112 clinical specimens (45 throat and 67 rectal swabs from 112 children) were used. Sequencing of VP1 PCR products was done using the BigDye Terminator V3.1 Cycle Sequencing kit in an ABI 3130XL DNA sequencer (Applied Biosystems, Carlsbad, CA, USA). The sequences were assembled using ContigExpress software - a component of Vector NTI Suit 7 (Informax Inc., NY, USA). Viral serotype was determined as described previously [13], e.g. by BLASTing the obtained sequences against the sequence available in GenBank.

### Assay application

**Fig. 4:**
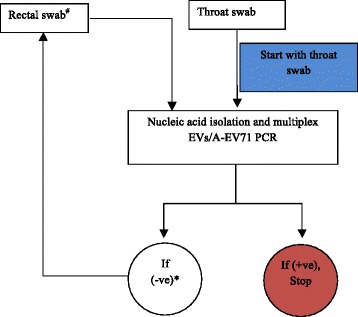
A flow chart showing the work-flow diagnosis. Note: +ve = positive; -ve = negative ^#^ Rectal swab analysis only when patients had throat-swab PCR negative *The analysis ended when rectal swab PCR is completed

The assay was applied on clinical samples obtained from 322 children with clinically suspected HFMD enrolled to our ongoing HFMD research program. The analysis was carried out using the diagnostic work-flow as described in Fig. 4, which includes an initial screening for EVs/EV-A71 in throat swabs and, to maximize the yield of the detection, rectal swabs from children who had a negative throat swab (Fig. 4) was further tested.

### Ethical approval

Clinical specimens used in the present study were derived from our ongoing HFMD research program conducted at HTD, CH1 and CH2. These studies were reviewed and approved by the local Institutional Review Boards and the Oxford Tropical Research Ethical Committee (OxTREC). Informed consent was obtained from parents or legal guardians of all enrolled children.

## Additional file

Additional file 1: Figure S1. A snapshot showing multiple sequence alignment and EV-A71 primer/probe binding sites.
