# Supplementary material for: Validation and utilization of an internally controlled multiplex Real-time RT-PCR assay for simultaneous detection of enteroviruses and enterovirus A71 associated with hand foot and mouth disease
Source: Virol J. 2015 Jun 9;12:85. doi: 10.1186/s12985-015-0316-2 (PMC4464700; doi:10.1186/s12985-015-0316-2)
Supplement: Additional file 1: Figure S1. — A snapshot showing multiple sequence alignment and EV-A71 primer/probe binding sites. [file 12985_2015_316_MOESM1_ESM.pdf]

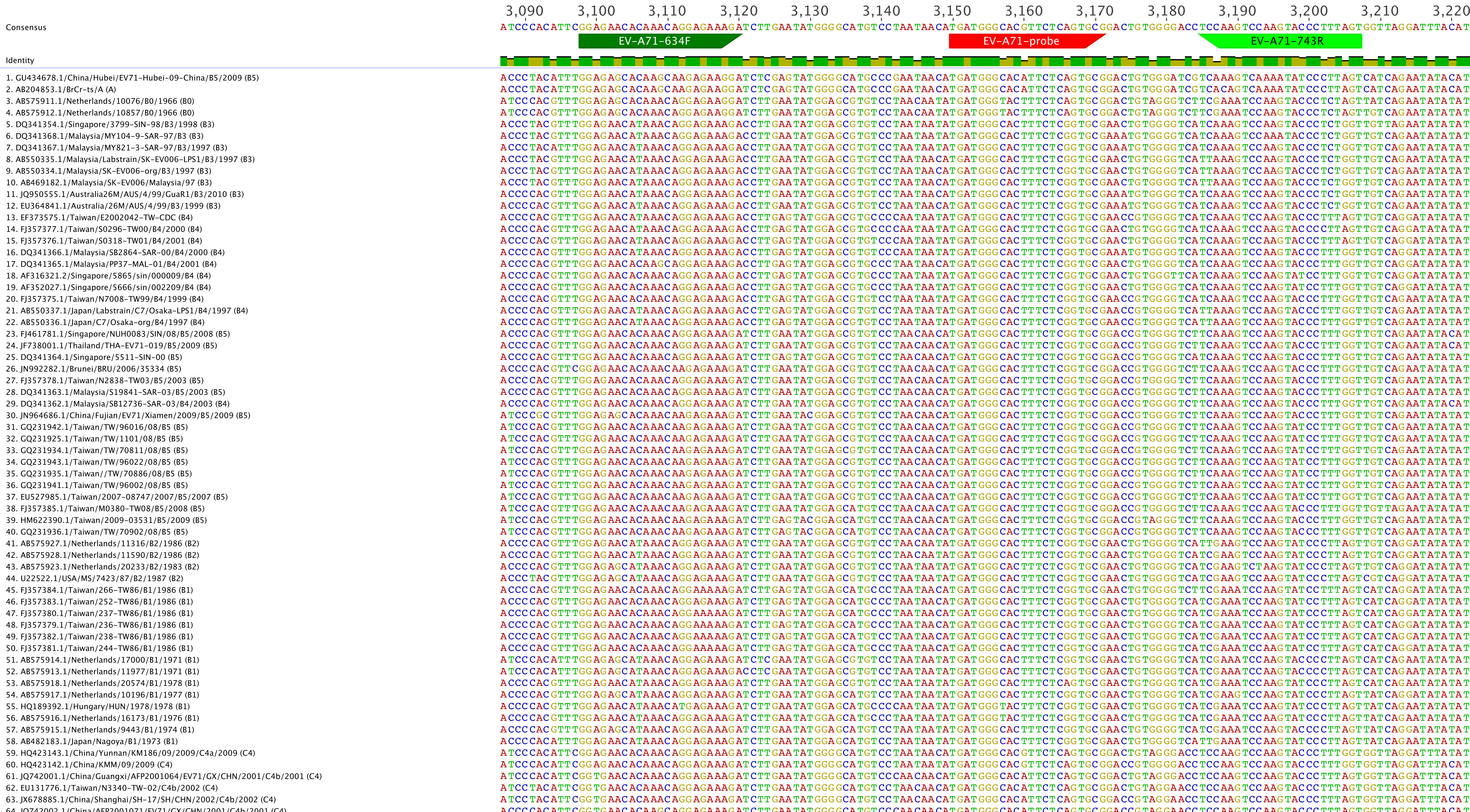

3,090
3,100
3,110
3,120
3,130
3,140
3,150
3,160
3,170
3,180
3,190
3,200
3,210
3,220
Consensus
EV-A71-634F
EV-A71-probe
EV-A71-743R
Identity
1. GU434678.1/China/Hubei/EV71-Hubei-09-China/B5/2009 (B5)
2. AB204853.1/BrCr-ts/A (A)
3. AB575911.1/Netherlands/10076/B0/1966 (B0)
4. AB575912.1/Netherlands/10857/B0/1966 (B0)
5. DQ341354.1/Singapore/3799-SIN-98/B3/1998 (B3)
6. DQ341368.1/Malaysia/MY104-9-SAR-97/B3 (B3)
7. DQ341367.1/Malaysia/MY821-3-SAR-97/B3/1997 (B3)
8. AB550335.1/Malaysia/Labstrain/SK-EV006-LPS1/B3/1997 (B3)
9. AB550334.1/Malaysia/SK-EV006-org/B3/1997 (B3)
10. AB469182.1/Malaysia/SK-EV006/Malaysia/97 (B3)
11. JQ950555.1/Australia26M/AUS/4/99/GuaR1/B3/2010 (B3)
12. EU364841.1/Australia/26M/AUS/4/99/B3/1999 (B3)
13. EF373575.1/Taiwan/E2002042-TW-CDC (B4)
14. FJ357377.1/Taiwan/S0296-TW00/B4/2000 (B4)
15. FJ357376.1/Taiwan/S0318-TW01/B4/2001 (B4)
16. DQ341366.1/Malaysia/SB2864-SAR-00/B4/2000 (B4)
17. DQ341365.1/Malaysia/PP37-MAL-01/B4/2001 (B4)
18. AF316321.2/Singapore/5865/sin/000009/B4 (B4)
19. AF352027.1/Singapore/5666/sin/002209/B4 (B4)
20. FJ357375.1/Taiwan/N7008-TW99/B4/1999 (B4)
21. AB550337.1/Japan/Labstrain/C7/Osaka-LPS1/B4/1997 (B4)
22. AB550336.1/Japan/C7/Osaka-org/B4/1997 (B4)
23. FJ461781.1/Singapore/NUH0083/SIN/08/B5/2008 (B5)
24. JF738001.1/Thailand/THA-EV71-019/B5/2009 (B5)
25. DQ341364.1/Singapore/5511-SIN-00 (B5)
26. JN992282.1/Brunei/BRU/2006/35334 (B5)
27. FJ357378.1/Taiwan/N2838-TW03/B5/2003 (B5)
28. DQ341363.1/Malaysia/S19841-SAR-03/B5/2003 (B5)
29. DQ341362.1/Malaysia/SB12736-SAR-03/B4/2003 (B4)
30. JN964686.1/China/Fujian/EV71/Xiamen/2009/B5/2009 (B5)
31. GQ231942.1/Taiwan/TW/96016/08/B5 (B5)
32. GQ231925.1/Taiwan/TW/1101/08/B5 (B5)
33. GQ231934.1/Taiwan/TW/70811/08/B5 (B5)
34. GQ231943.1/Taiwan/TW/96022/08/B5 (B5)
35. GQ231935.1/Taiwan//TW/70886/08/B5 (B5)
36. GQ231941.1/Taiwan/TW/96002/08/B5 (B5)
37. EU527985.1/Taiwan/2007-08747/2007/B5/2007 (B5)
38. FJ357385.1/Taiwan/M0380-TW08/B5/2008 (B5)
39. HM622390.1/Taiwan/2009-03531/B5/2009 (B5)
40. GQ231936.1/Taiwan/TW/70902/08/B5 (B5)
41. AB575927.1/Netherlands/11316/B2/1986 (B2)
42. AB575928.1/Netherlands/11590/B2/1986 (B2)
43. AB575923.1/Netherlands/20233/B2/1983 (B2)
44. U22522.1/USA/MS/7423/87/B2/1987 (B2)
45. FJ357384.1/Taiwan/266-TW86/B1/1986 (B1)
46. FJ357383.1/Taiwan/252-TW86/B1/1986 (B1)
47. FJ357380.1/Taiwan/237-TW86/B1/1986 (B1)
48. FJ357379.1/Taiwan/236-TW86/B1/1986 (B1)
49. FJ357382.1/Taiwan/238-TW86/B1/1986 (B1)
50. FJ357381.1/Taiwan/244-TW86/B1/1986 (B1)
51. AB575914.1/Netherlands/17000/B1/1971 (B1)
52. AB575913.1/Netherlands/11977/B1/1971 (B1)
53. AB575918.1/Netherlands/20574/B1/1978 (B1)
54. AB575917.1/Netherlands/10196/B1/1977 (B1)
55. HQ189392.1/Hungary/HUN/1978/1978 (B1)
56. AB575916.1/Netherlands/16173/B1/1976 (B1)
57. AB575915.1/Netherlands/9443/B1/1974 (B1)
58. AB482183.1/Japan/Nagoya/B1/1973 (B1)
59. HQ423143.1/China/Yunnan/KM186/09/2009/C4a/2009 (C4)
60. HQ423142.1/China/KMM/09/2009 (C4)
61. JQ742001.1/China/Guangxi/AFP2001064/EV71/GX/CHN/2001/C4b/2001 (C4)
62. EU131776.1/Taiwan/N3340-TW-02/C4b/2002 (C4)
63. JX678885.1/China/Shanghai/SH-17/SH/CHN/2002/C4b/2002 (C4)

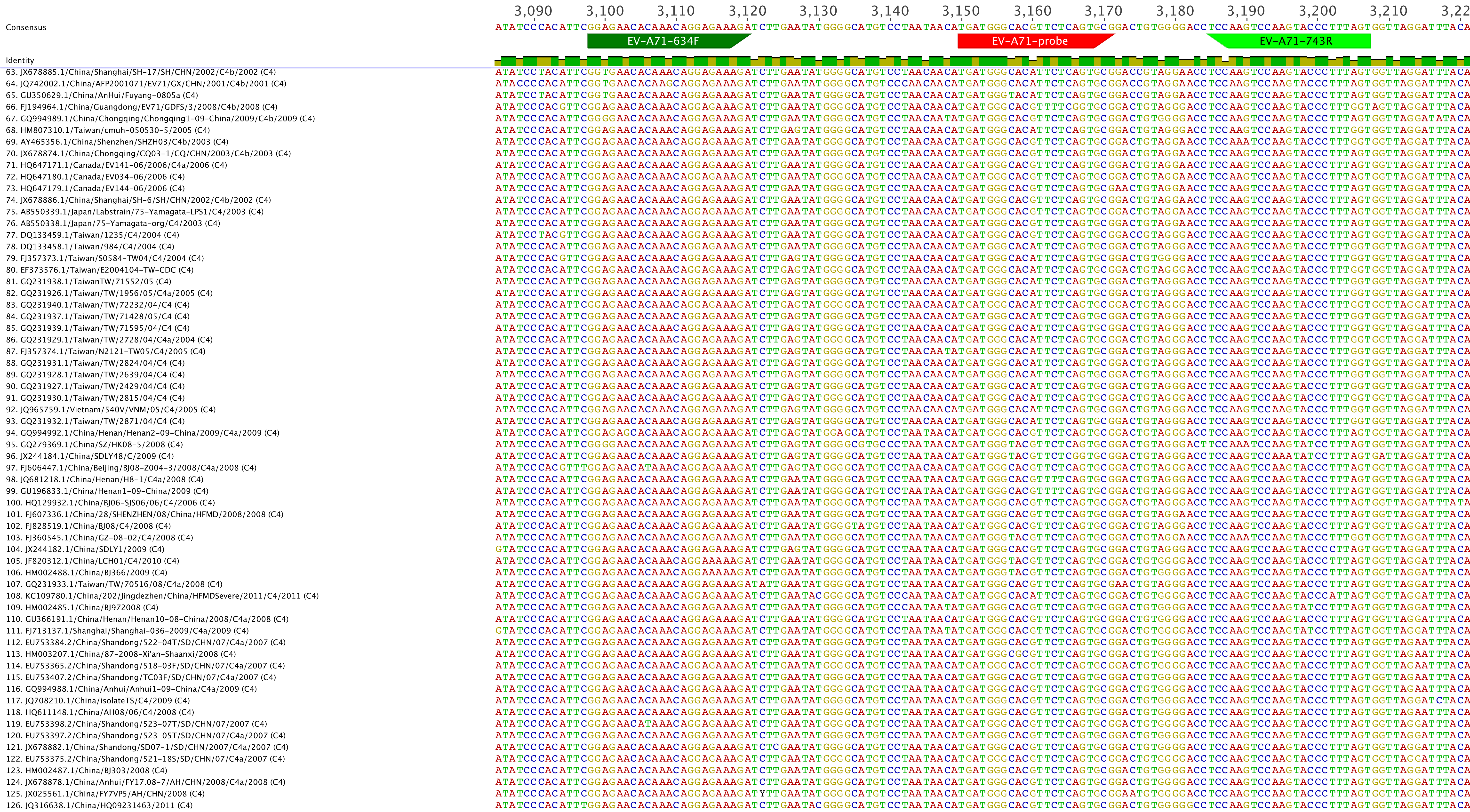
3,090 3,100 3,110 3,120 3,130 3,140 3,150 3,160 3,170 3,180 3,190 3,200 3,210 3,22
Consensus
ATATCCCACATTCGGAGAACACAAACAGGAGAAAGATCTTGAATATGGGGCATGTCCTAATAACATGATGGGCACGTTCTCAGTGCGGACTGTGGGGACCTCCAAGTCCAAGTACCCTTTAGTGGTTAGGATTTACA
EV-A71-634F
EV-A71-probe
EV-A71-743R
Identity
63. JX678885.1/China/Shanghai/SH-17/SH/CHN/2002/C4b/2002 (C4)
64. JQ742002.1/China/AFP2001071/EV71/GX/CHN/2001/C4b/2001 (C4)
65. GU350629.1/China/AnHui/Fuyang-0805a (C4)
66. FJ194964.1/China/Guangdong/EV71/GDFS/3/2008/C4b/2008 (C4)
67. GQ994989.1/China/Chongqing/Chongqing1-09-China/2009/C4b/2009 (C4)
68. HM807310.1/Taiwan/cmuh-050530-5/2005 (C4)
69. AY465356.1/China/Shenzhen/SHZH03/C4b/2003 (C4)
70. JX678874.1/China/Chongqing/CQ03-1/CQ/CHN/2003/C4b/2003 (C4)
71. HQ647171.1/Canada/EV141-06/2006/C4a/2006 (C4)
72. HQ647180.1/Canada/EV034-06/2006 (C4)
73. HQ647179.1/Canada/EV144-06/2006 (C4)
74. JX678886.1/China/Shanghai/SH-6/SH/CHN/2002/C4b/2002 (C4)
75. AB550339.1/Japan/Labstrain/75-Yamagata-LPS1/C4/2003 (C4)
76. AB550338.1/Japan/75-Yamagata-org/C4/2003 (C4)
77. DQ133459.1/Taiwan/1235/C4/2004 (C4)
78. DQ133458.1/Taiwan/984/C4/2004 (C4)
79. FJ357373.1/Taiwan/S0584-TW04/C4/2004 (C4)
80. EF373576.1/Taiwan/E2004104-TW-CDC (C4)
81. GQ231938.1/TaiwanTW/71552/05 (C4)
82. GQ231926.1/Taiwan/TW/1956/05/C4a/2005 (C4)
83. GQ231940.1/Taiwan/TW/72232/04/C4 (C4)
84. GQ231937.1/Taiwan/TW/71428/05/C4 (C4)
85. GQ231939.1/Taiwan/TW/71595/04/C4 (C4)
86. GQ231929.1/Taiwan/TW/2728/04/C4a/2004 (C4)
87. FJ357374.1/Taiwan/N2121-TW05/C4/2005 (C4)
88. GQ231931.1/Taiwan/TW/2824/04/C4 (C4)
89. GQ231928.1/Taiwan/TW/2639/04/C4 (C4)
90. GQ231927.1/Taiwan/TW/2429/04/C4 (C4)
91. GQ231930.1/Taiwan/TW/2815/04/C4 (C4)
92. JQ965759.1/Vietnam/540V/VNM/05/C4/2005 (C4)
93. GQ231932.1/Taiwan/TW/2871/04/C4 (C4)
94. GQ994992.1/China/Henan/Henan2-09-China/2009/C4a/2009 (C4)
95. GQ279369.1/China/SZ/HK08-5/2008 (C4)
96. JX244184.1/China/SDLY48/C/2009 (C4)
97. FJ606447.1/China/Beijing/BJ08-Z004-3/2008/C4a/2008 (C4)
98. JQ681218.1/China/Henan/H8-1/C4a/2008 (C4)
99. GU196833.1/China/Henan1-09-China/2009 (C4)
100. HQ129932.1/China/BJ06-SJS06/06/C4/2006 (C4)
101. FJ607336.1/China/28/SHENZHEN/08/China/HFMD/2008/2008 (C4)
102. FJ828519.1/China/BJ08/C4/2008 (C4)
103. FJ360545.1/China/GZ-08-02/C4/2008 (C4)
104. JX244182.1/China/SDLY1/2009 (C4)
105. JF820312.1/China/LCH01/C4/2010 (C4)
106. HM002488.1/China/BJ366/2009 (C4)
107. GQ231933.1/Taiwan/TW/70516/08/C4a/2008 (C4)
108. KC109780.1/China/202/Jingdezhen/China/HFMDSevere/2011/C4/2011 (C4)
109. HM002485.1/China/BJ972008 (C4)
110. GU366191.1/China/Henan/Henan10-08-China/2008/C4a/2008 (C4)
111. FJ713137.1/Shanghai/Shanghai-036-2009/C4a/2009 (C4)
112. EU753384.2/China/Shandong/522-04T/SD/CHN/07/C4a/2007 (C4)
113. HM003207.1/China/87-2008-Xi'an-Shaanxi/2008 (C4)
114. EU753365.2/China/Shandong/518-03F/SD/CHN/07/C4a/2007 (C4)
115. EU753407.2/China/Shandong/TC03F/SD/CHN/07/C4a/2007 (C4)
116. GQ994988.1/China/Anhui/Anhui1-09-China/C4a/2009 (C4)
117. JQ708210.1/China/isolateTS/C4/2009 (C4)
118. HQ611148.1/China/AH08/06/C4/2008 (C4)
119. EU753398.2/China/Shandong/523-07T/SD/CHN/07/2007 (C4)
120. EU753397.2/China/Shandong/523-05T/SD/CHN/07/C4a/2007 (C4)
121. JX678882.1/China/Shandong/SD07-1/SD/CHN/2007/C4a/2007 (C4)
122. EU753375.2/China/Shandong/521-18S/SD/CHN/07/C4a/2007 (C4)
123. HM002487.1/China/BJ303/2008 (C4)
124. JX678878.1/China/Anhui/FY17.08-7/AH/CHN/2008/C4a/2008 (C4)
125. JX025561.1/China/FY7VP5/AH/CHN/2008 (C4)
126. JQ316638.1/China/HQ09231463/2011 (C4)

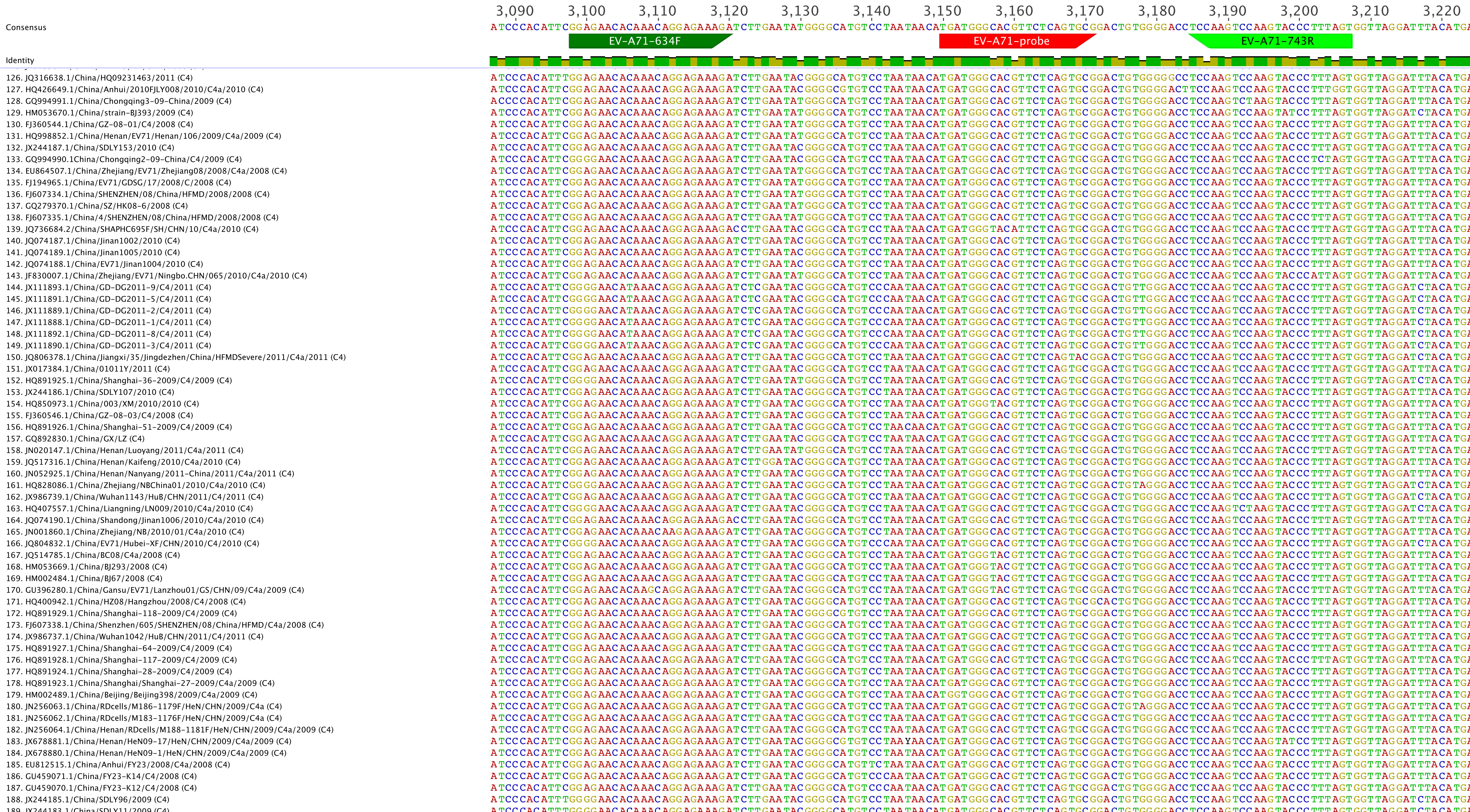
3,090 3,100 3,110 3,120 3,130 3,140 3,150 3,160 3,170 3,180 3,190 3,200 3,210 3,220
Consensus
EV-A71-634F
EV-A71-probe
EV-A71-743R
Identity
126. JQ316638.1/China/HQ09231463/2011 (C4)
127. HQ426649.1/China/Anhui/2010FJLY008/2010/C4a/2010 (C4)
128. GQ994991.1/China/Chongqing3-09-China/2009 (C4)
129. HM053670.1/China/strain-BJ393/2009 (C4)
130. FJ360544.1/China/GZ-08-01/C4/2008 (C4)
131. HQ998852.1/China/Henan/EV71/Henan/106/2009/C4a/2009 (C4)
132. JX244187.1/China/SDLY153/2010 (C4)
133. GQ994990.1China/Chongqing2-09-China/C4/2009 (C4)
134. EU864507.1/China/Zhejiang/EV71/Zhejiang08/2008/C4a/2008 (C4)
135. FJ194965.1/China/EV71/GDSG/17/2008/C/2008 (C4)
136. FJ607334.1/China/SHENZHEN/08/China/HFMD/2008/2008 (C4)
137. GQ279370.1/China/SZ/HK08-6/2008 (C4)
138. FJ607335.1/China/4/SHENZHEN/08/China/HFMD/2008/2008 (C4)
139. JQ736684.2/China/SHAPHC695F/SH/CHN/10/C4a/2010 (C4)
140. JQ074187.1/China/Jinan1002/2010 (C4)
141. JQ074189.1/China/Jinan1005/2010 (C4)
142. JQ074188.1/China/EV71/Jinan1004/2010 (C4)
143. JF830007.1/China/Zhejiang/EV71/Ningbo.CHN/065/2010/C4a/2010 (C4)
144. JX111893.1/China/GD-DG2011-9/C4/2011 (C4)
145. JX111891.1/China/GD-DG2011-5/C4/2011 (C4)
146. JX111889.1/China/GD-DG2011-2/C4/2011 (C4)
147. JX111888.1/China/GD-DG2011-1/C4/2011 (C4)
148. JX111892.1/China/GD-DG2011-8/C4/2011 (C4)
149. JX111890.1/China/GD-DG2011-3/C4/2011 (C4)
150. JQ806378.1/China/Jiangxi/35/Jingdezhen/China/HFMDSevere/2011/C4a/2011 (C4)
151. JX017384.1/China/01011Y/2011 (C4)
152. HQ891925.1/China/Shanghai-36-2009/C4/2009 (C4)
153. JX244186.1/China/SDLY107/2010 (C4)
154. HQ850973.1/China/003/XM/2010/2010 (C4)
155. FJ360546.1/China/GZ-08-03/C4/2008 (C4)
156. HQ891926.1/China/Shanghai-51-2009/C4/2009 (C4)
157. GQ892830.1/China/GX/LZ (C4)
158. JN020147.1/China/Henan/Luoyang/2011/C4a/2011 (C4)
159. JQ517316.1/China/Henan/Kaifeng/2010/C4a/2010 (C4)
160. JN052925.1/China/Henan/Nanyang/2011-China/2011/C4a/2011 (C4)
161. HQ828086.1/China/Zhejiang/NBChina01/2010/C4a/2010 (C4)
162. JX986739.1/China/Wuhan1143/HuB/CHN/2011/C4/2011 (C4)
163. HQ407557.1/China/Liangning/LN009/2010/C4a/2010 (C4)
164. JQ074190.1/China/Shandong/Jinan1006/2010/C4a/2010 (C4)
165. JN001860.1/China/Zhejiang/NB/2010/01/C4a/2010 (C4)
166. JQ804832.1/China/EV71/Hubei-XF/CHN/2010/C4/2010 (C4)
167. JQ514785.1/China/BC08/C4a/2008 (C4)
168. HM053669.1/China/BJ293/2008 (C4)
169. HM002484.1/China/BJ67/2008 (C4)
170. GU396280.1/China/Gansu/EV71/Lanzhou01/GS/CHN/09/C4a/2009 (C4)
171. HQ400942.1/China/HZ08/Hangzhou/2008/C4/2008 (C4)
172. HQ891929.1/China/Shanghai-118-2009/C4/2009 (C4)
173. FJ607338.1/China/Shenzhen/605/SHENZHEN/08/China/HFMD/C4a/2008 (C4)
174. JX986737.1/China/Wuhan1042/HuB/CHN/2011/C4/2011 (C4)
175. HQ891927.1/China/Shanghai-64-2009/C4/2009 (C4)
176. HQ891928.1/China/Shanghai-117-2009/C4/2009 (C4)
177. HQ891924.1/China/Shanghai-28-2009/C4/2009 (C4)
178. HQ891923.1/China/Shanghai/Shanghai-27-2009/C4a/2009 (C4)
179. HM002489.1/China/Beijing/Beijing398/2009/C4a/2009 (C4)
180. JN256063.1/China/RDcells/M186-1179F/HeN/CHN/2009/C4a (C4)
181. JN256062.1/China/RDcells/M183-1176F/HeN/CHN/2009/C4a (C4)
182. JN256064.1/China/Henan/RDcells/M188-1181F/HeN/CHN/2009/C4a/2009 (C4)
183. JX678881.1/China/Henan/HeN09-17/HeN/CHN/2009/C4a/2009 (C4)
184. JX678880.1/China/Henan/HeN09-1/HeN/CHN/2009/C4a/2009 (C4)
185. EU812515.1/China/Anhui/FY23/2008/C4a/2008 (C4)
186. GU459071.1/China/FY23-K14/C4/2008 (C4)
187. GU459070.1/China/FY23-K12/C4/2008 (C4)
188. JX244185.1/China/SDLY96/2009 (C4)

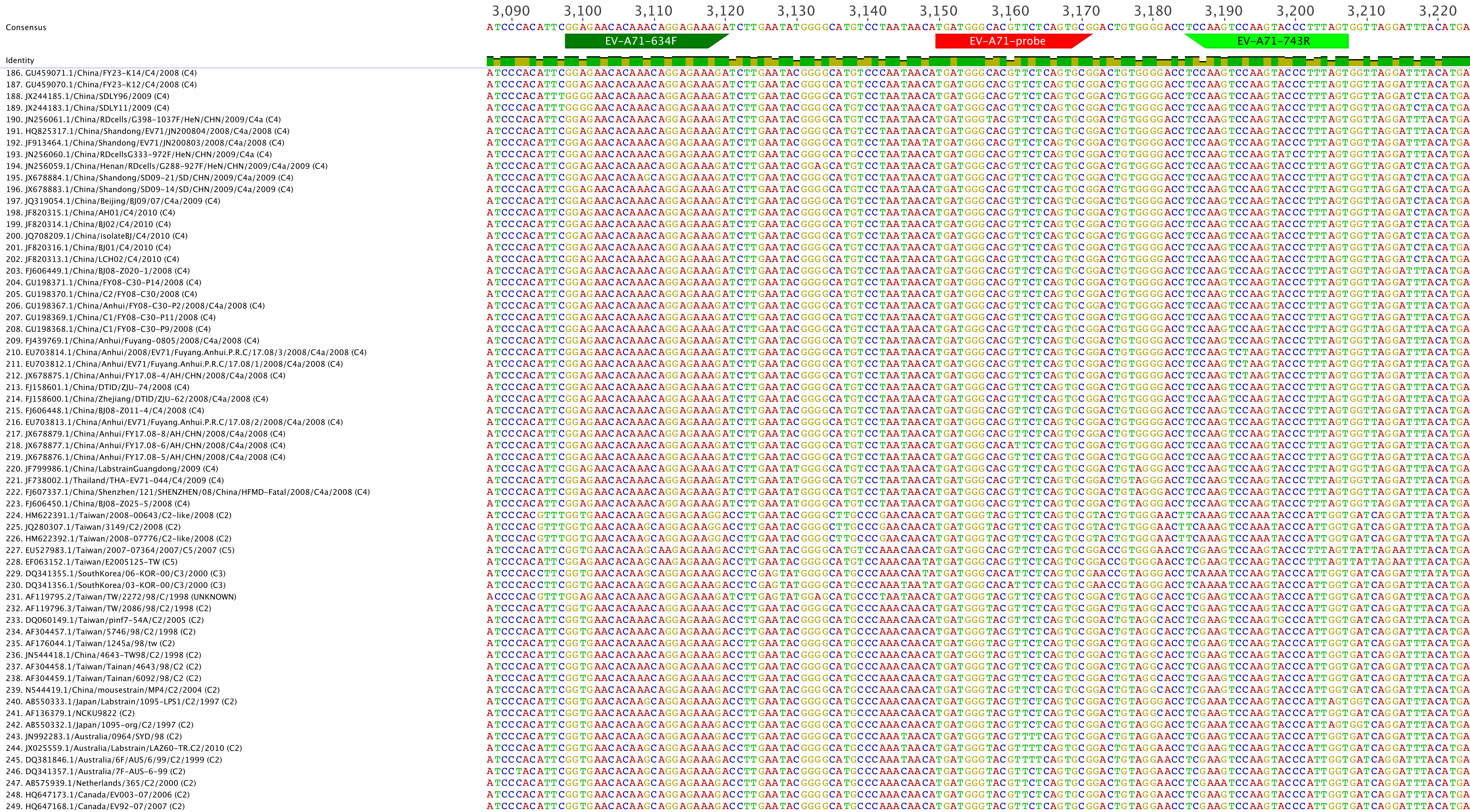
3,090
3,100
3,110
3,120
3,130
3,140
3,150
3,160
3,170
3,180
3,190
3,200
3,210
3,220
Consensus
EV-A71-634F
EV-A71-probe
EV-A71-743R
Identity
186. GU459071.1/China/FY23-K14/C4/2008 (C4)
187. GU459070.1/China/FY23-K12/C4/2008 (C4)
188. JX244185.1/China/SDLY96/2009 (C4)
189. JX244183.1/China/SDLY11/2009 (C4)
190. JN256061.1/China/RDcells/G398-1037F/HeN/CHN/2009/C4a (C4)
191. HQ825317.1/China/Shandong/EV71/JN200804/2008/C4a/2008 (C4)
192. JF913464.1/China/Shandong/EV71/JN200803/2008/C4a/2008 (C4)
193. JN256060.1/China/RDcellsG333-972F/HeN/CHN/2009/C4a (C4)
194. JN256059.1/China/Henan/RDcells/G288-927F/HeN/CHN/2009/C4a/2009 (C4)
195. JX678884.1/China/Shandong/SD09-21/SD/CHN/2009/C4a/2009 (C4)
196. JX678883.1/China/Shandong/SD09-14/SD/CHN/2009/C4a/2009 (C4)
197. JQ319054.1/China/Beijing/BJ09/07/C4a/2009 (C4)
198. JF820315.1/China/AH01/C4/2010 (C4)
199. JF820314.1/China/BJ02/C4/2010 (C4)
200. JQ708209.1/China/isolateBJ/C4/2010 (C4)
201. JF820316.1/China/BJ01/C4/2010 (C4)
202. JF820313.1/China/LCH02/C4/2010 (C4)
203. FJ606449.1/China/BJ08-Z020-1/2008 (C4)
204. GU198371.1/China/FY08-C30-P14/2008 (C4)
205. GU198370.1/China/C2/FY08-C30/2008 (C4)
206. GU198367.1/China/Anhui/FY08-C30-P2/2008/C4a/2008 (C4)
207. GU198369.1/China/C1/FY08-C30-P11/2008 (C4)
208. GU198368.1/China/C1/FY08-C30-P9/2008 (C4)
209. FJ439769.1/China/Anhui/Fuyang-0805/2008/C4a/2008 (C4)
210. EU703814.1/China/Anhui/2008/EV71/Fuyang.Anhui.P.R.C/17.08/3/2008/C4a/2008 (C4)
211. EU703812.1/China/Anhui/EV71/Fuyang.Anhui.P.R.C/17.08/1/2008/C4a/2008 (C4)
212. JX678875.1/China/Anhui/FY17.08-4/AH/CHN/2008/C4a/2008 (C4)
213. FJ158601.1/China/DTID/ZJU-74/2008 (C4)
214. FJ158600.1/China/Zhejiang/DTID/ZJU-62/2008/C4a/2008 (C4)
215. FJ606448.1/China/BJ08-Z011-4/C4/2008 (C4)
216. EU703813.1/China/Anhui/EV71/Fuyang.Anhui.P.R.C/17.08/2/2008/C4a/2008 (C4)
217. JX678879.1/China/Anhui/FY17.08-8/AH/CHN/2008/C4a/2008 (C4)
218. JX678877.1/China/Anhui/FY17.08-6/AH/CHN/2008/C4a/2008 (C4)
219. JX678876.1/China/Anhui/FY17.08-5/AH/CHN/2008/C4a/2008 (C4)
220. JF799986.1/China/LabstrainGuangdong/2009 (C4)
221. JF738002.1/Thailand/THA-EV71-044/C4/2009 (C4)
222. FJ607337.1/China/Shenzhen/121/SHENZHEN/08/China/HFMD-Fatal/2008/C4a/2008 (C4)
223. FJ606450.1/China/BJ08-Z025-5/2008 (C4)
224. HM622391.1/Taiwan/2008-00643/C2-like/2008 (C2)
225. JQ280307.1/Taiwan/3149/C2/2008 (C2)
226. HM622392.1/Taiwan/2008-07776/C2-like/2008 (C2)
227. EU527983.1/Taiwan/2007-07364/2007/C5/2007 (C5)
228. EF063152.1/Taiwan/E2005125-TW (C5)
229. DQ341355.1/SouthKorea/06-KOR-00/C3/2000 (C3)
230. DQ341356.1/SouthKorea/03-KOR-00/C3/2000 (C3)
231. AF119795.2/Taiwan/TW/2272/98/C/1998 (UNKNOWN)
232. AF119796.3/Taiwan/TW/2086/98/C2/1998 (C2)
233. DQ060149.1/Taiwan/pinf7-54A/C2/2005 (C2)
234. AF304457.1/Taiwan/5746/98/C2/1998 (C2)
235. AF176044.1/Taiwan/1245a/98/tw (C2)
236. JN544418.1/China/4643-TW98/C2/1998 (C2)
237. AF304458.1/Taiwan/Tainan/4643/98/C2 (C2)
238. AF304459.1/Taiwan/Tainan/6092/98/C2 (C2)
239. N544419.1/China/mousestrain/MP4/C2/2004 (C2)
240. AB550333.1/Japan/Labstrain/1095-LPS1/C2/1997 (C2)
241. AF136379.1/NCKU9822 (C2)
242. AB550332.1/Japan/1095-org/C2/1997 (C2)
243. JN992283.1/Australia/0964/SYD/98 (C2)
244. JX025559.1/Australia/Labstrain/LAZ60-TR.C2/2010 (C2)
245. DQ381846.1/Australia/6F/AUS/6/99/C2/1999 (C2)
246. DQ341357.1/Australia/7F-AUS-6-99 (C2)
247. AB575939.1/Netherlands/365/C2/2000 (C2)
248. HQ647173.1/Canada/EV003-07/2006 (C2)
249. HQ647168.1/Canada/EV92-07/2007 (C2)

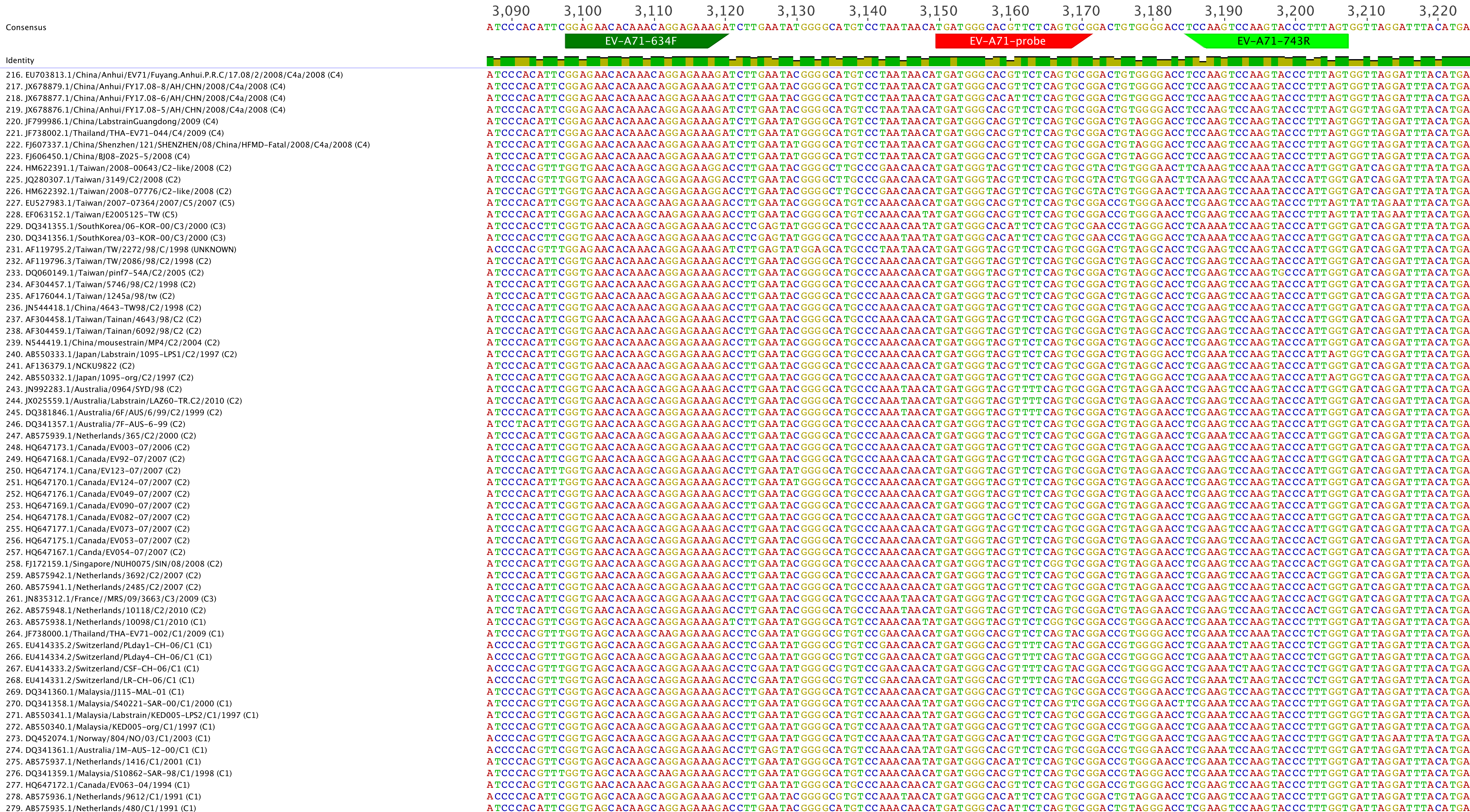

3,090
3,100
3,110
3,120
3,130
3,140
3,150
3,160
3,170
3,180
3,190
3,200
3,210
3,220
Consensus
EV-A71-634F
EV-A71-probe
EV-A71-743R
Identity
216. EU703813.1/China/Anhui/EV71/Fuyang.Anhui.P.R.C/17.08/2/2008/C4a/2008 (C4)
217. JX678879.1/China/Anhui/FY17.08-8/AH/CHN/2008/C4a/2008 (C4)
218. JX678877.1/China/Anhui/FY17.08-6/AH/CHN/2008/C4a/2008 (C4)
219. JX678876.1/China/Anhui/FY17.08-5/AH/CHN/2008/C4a/2008 (C4)
220. JF799986.1/China/LabstrainGuangdong/2009 (C4)
221. JF738002.1/Thailand/THA-EV71-044/C4/2009 (C4)
222. FJ607337.1/China/Shenzhen/121/SHENZHEN/08/China/HFMD-Fatal/2008/C4a/2008 (C4)
223. FJ606450.1/China/BJ08-Z025-5/2008 (C4)
224. HM622391.1/Taiwan/2008-00643/C2-like/2008 (C2)
225. JQ280307.1/Taiwan/3149/C2/2008 (C2)
226. HM622392.1/Taiwan/2008-07776/C2-like/2008 (C2)
227. EU527983.1/Taiwan/2007-07364/2007/C5/2007 (C5)
228. EF063152.1/Taiwan/E2005125-TW (C5)
229. DQ341355.1/SouthKorea/06-KOR-00/C3/2000 (C3)
230. DQ341356.1/SouthKorea/03-KOR-00/C3/2000 (C3)
231. AF119795.2/Taiwan/TW/2272/98/C/1998 (UNKNOWN)
232. AF119796.3/Taiwan/TW/2086/98/C2/1998 (C2)
233. DQ060149.1/Taiwan/pinf7-54A/C2/2005 (C2)
234. AF304457.1/Taiwan/5746/98/C2/1998 (C2)
235. AF176044.1/Taiwan/1245a/98/tw (C2)
236. JN544418.1/China/4643-TW98/C2/1998 (C2)
237. AF304458.1/Taiwan/Tainan/4643/98/C2 (C2)
238. AF304459.1/Taiwan/Tainan/6092/98/C2 (C2)
239. N544419.1/China/mousestrain/MP4/C2/2004 (C2)
240. AB550333.1/Japan/Labstrain/1095-LPS1/C2/1997 (C2)
241. AF136379.1/NCKU9822 (C2)
242. AB550332.1/Japan/1095-org/C2/1997 (C2)
243. JN992283.1/Australia/0964/SYD/98 (C2)
244. JX025559.1/Australia/Labstrain/LAZ60-TR.C2/2010 (C2)
245. DQ381846.1/Australia/6F/AUS/6/99/C2/1999 (C2)
246. DQ341357.1/Australia/7F-AUS-6-99 (C2)
247. AB575939.1/Netherlands/365/C2/2000 (C2)
248. HQ647173.1/Canada/EV003-07/2006 (C2)
249. HQ647168.1/Canada/EV92-07/2007 (C2)
250. HQ647174.1/Cana/EV123-07/2007 (C2)
251. HQ647170.1/Canada/EV124-07/2007 (C2)
252. HQ647176.1/Canada/EV049-07/2007 (C2)
253. HQ647169.1/Canada/EV090-07/2007 (C2)
254. HQ647178.1/Canada/EV082-07/2007 (C2)
255. HQ647177.1/Canada/EV073-07/2007 (C2)
256. HQ647175.1/Canada/EV053-07/2007 (C2)
257. HQ647167.1/Canda/EV054-07/2007 (C2)
258. FJ172159.1/Singapore/NUH0075/SIN/08/2008 (C2)
259. AB575942.1/Netherlands/3692/C2/2007 (C2)
260. AB575941.1/Netherlands/2485/C2/2007 (C2)
261. JN835312.1/France//MRS/09/3663/C3/2009 (C3)
262. AB575948.1/Netherlands/10118/C2/2010 (C2)
263. AB575938.1/Netherlands/10098/C1/2010 (C1)
264. JF738000.1/Thailand/THA-EV71-002/C1/2009 (C1)
265. EU414335.2/Switzerland/PLday1-CH-06/C1 (C1)
266. EU414334.2/Switzerland/PLday4-CH-06/C1 (C1)
267. EU414333.2/Switzerland/CSF-CH-06/C1 (C1)
268. EU414331.2/Switzerland/LR-CH-06/C1 (C1)
269. DQ341360.1/Malaysia/J115-MAL-01 (C1)
270. DQ341358.1/Malaysia/S40221-SAR-00/C1/2000 (C1)
271. AB550341.1/Malaysia/Labstrain/KED005-LPS2/C1/1997 (C1)
272. AB550340.1/Malaysia/KED005-org/C1/1997 (C1)
273. DQ452074.1/Norway/804/NO/03/C1/2003 (C1)
274. DQ341361.1/Australia/1M-AUS-12-00/C1 (C1)
275. AB575937.1/Netherlands/1416/C1/2001 (C1)
276. DQ341359.1/Malaysia/S10862-SAR-98/C1/1998 (C1)
277. HQ647172.1/Canada/EV063-04/1994 (C1)
278. AB575936.1/Netherlands/9612/C1/1991 (C1)
279. AB575935.1/Netherlands/480/C1/1991 (C1)
